# Supplementary material for: A single-cell transcriptomic atlas of peripheral blood immune cells spanning progressive canine leishmaniosis
Source: bioRxiv. 2025 Dec 21:2025.12.18.695149. Preprint. [Version 1] doi: 10.64898/2025.12.18.695149 (PMC12724653; doi:10.64898/2025.12.18.695149)
Supplement: Supplement 1 [file media-1.pdf]

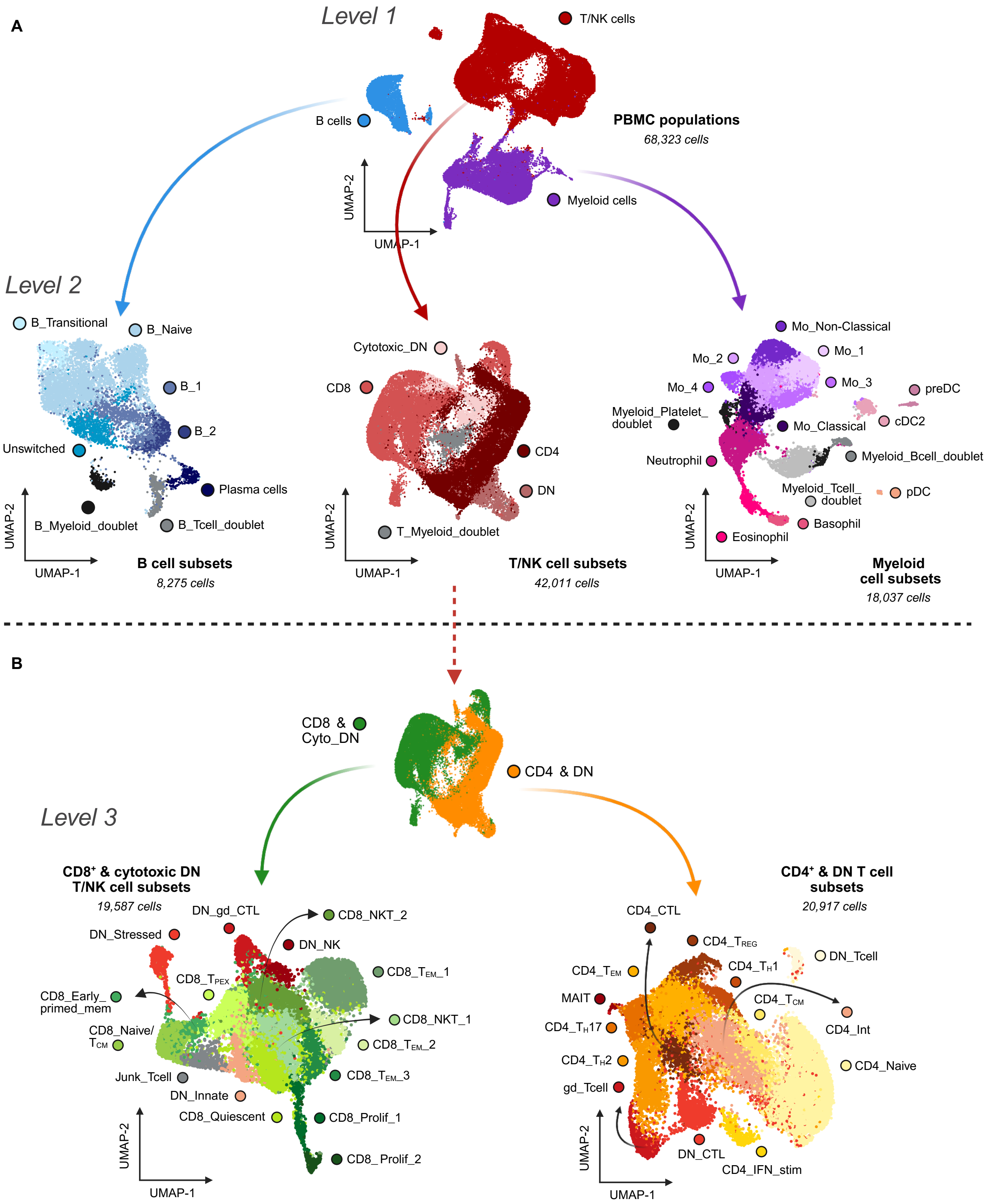

Figure S1: Strategy for stepwise immune subsetting.
Schematic of the subsetting strategy used to resolve major PBMC populations into Level 2 B, T/NK, and Myeloid subsets **(A)**; and T/NK cell populations into Level 3 CD4+ & DN and CD8+ & Cytotoxic DN subpopulations **(B)**.

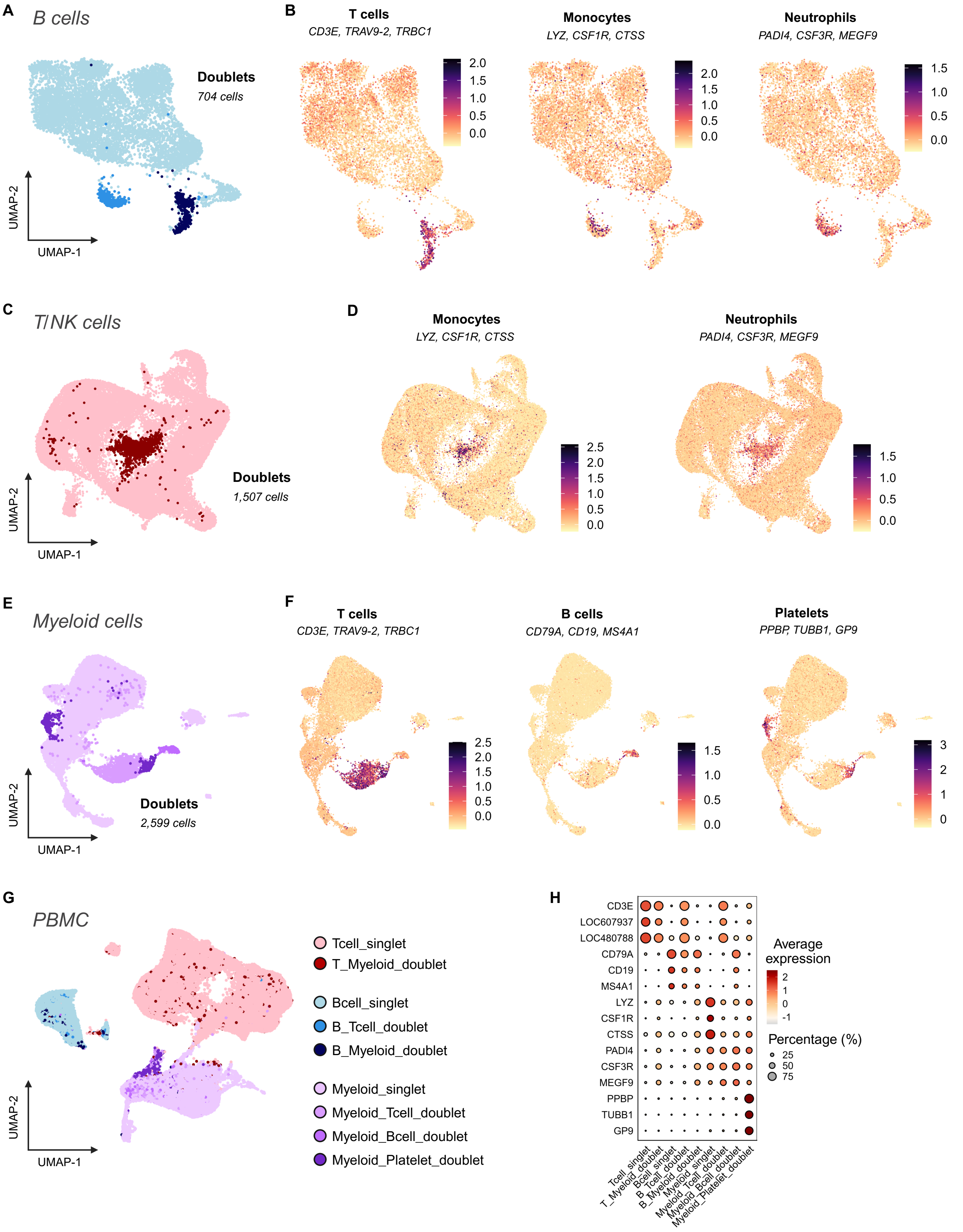
A
B cells
Doublets
704 cells
UMAP-2
UMAP-1
B
T cells
CD3E, TRAV9-2, TRBC1
Monocytes
LYZ, CSF1R, CTSS
Neutrophils
PADI4, CSF3R, MEGF9
C
T/NK cells
Doublets
1,507 cells
D
Monocytes
LYZ, CSF1R, CTSS
Neutrophils
PADI4, CSF3R, MEGF9
E
Myeloid cells
Doublets
2,599 cells
F
T cells
CD3E, TRAV9-2, TRBC1
B cells
CD79A, CD19, MS4A1
Platelets
PPBP, TUBB1, GP9
G
PBMC
Tcell_singlet
T_Myeloid_doublet
Bcell_singlet
B_Tcell_doublet
B_Myeloid_doublet
Myeloid_singlet
Myeloid_Tcell_doublet
Myeloid_Bcell_doublet
Myeloid_Platelet_doublet
H
CD3E
LOC607937
LOC480788
CD79A
CD19
MS4A1
LYZ
CSF1R
CTSS
PADI4
CSF3R
MEGF9
PPBP
TUBB1
GP9
Average expression
Percentage (%)
25
50
75

Figure S2: Co-expression of dual canonical markers for doublet identification.
(A) UMAP visualization showing B cell singlet and doublet populations within the Bcell object (see also Figure 2).
(B) Feature plots showing module scores for selected canonical marker genes used to identify and distinguish T cell (CD3E, LOC607937 (*TRAV9-1*); LOC480788 (*TRBC1*)), Monocyte (*LYZ*, *CSF1R*, *CTSS*) and Neutrophil (*PADI4*, *CSF3R*, *MEGF9*) populations in the Bcell object.
(C) UMAP visualization showing T cell singlet and doublet populations within the Tcell object (see also Figure 3).
(D) Feature plots showing module scores for selected canonical marker genes used to identify and distinguish Monocyte (*LYZ*, *CSF1R*, *CTSS*) and Neutrophil (*PADI4*, *CSF3R*, *MEGF9*) populations in the Tcell object.
(E) UMAP visualization showing Myeloid singlet and doublet populations within the myeloid object (see also Figure 6).
(F) Feature plots showing module scores for selected canonical marker genes used to identify and distinguish T cell (*CD3E*, *LOC607937* (*TRAV9*-1); *LOC480788* (*TRBC1*)), B cell (*CD79A*, *CD19*, *MS4A1*) and Platelet (*PPBP*, *TUBB1*, *GP9*) populations in the Myeloid object.
(**G**) UMAP visualization of the PBMC object displaying cells of the singlet and doublet populations. These were overlayed from the metadata of the level 2 (Myeloid, Tcell and Bcell) objects into level 1 (PBMC).
(**H**) Dot plot showing proportion and expression of canonical markers in singlet and doublet populations of the PBMC object generated as described in **B**, **D**, and **F**.

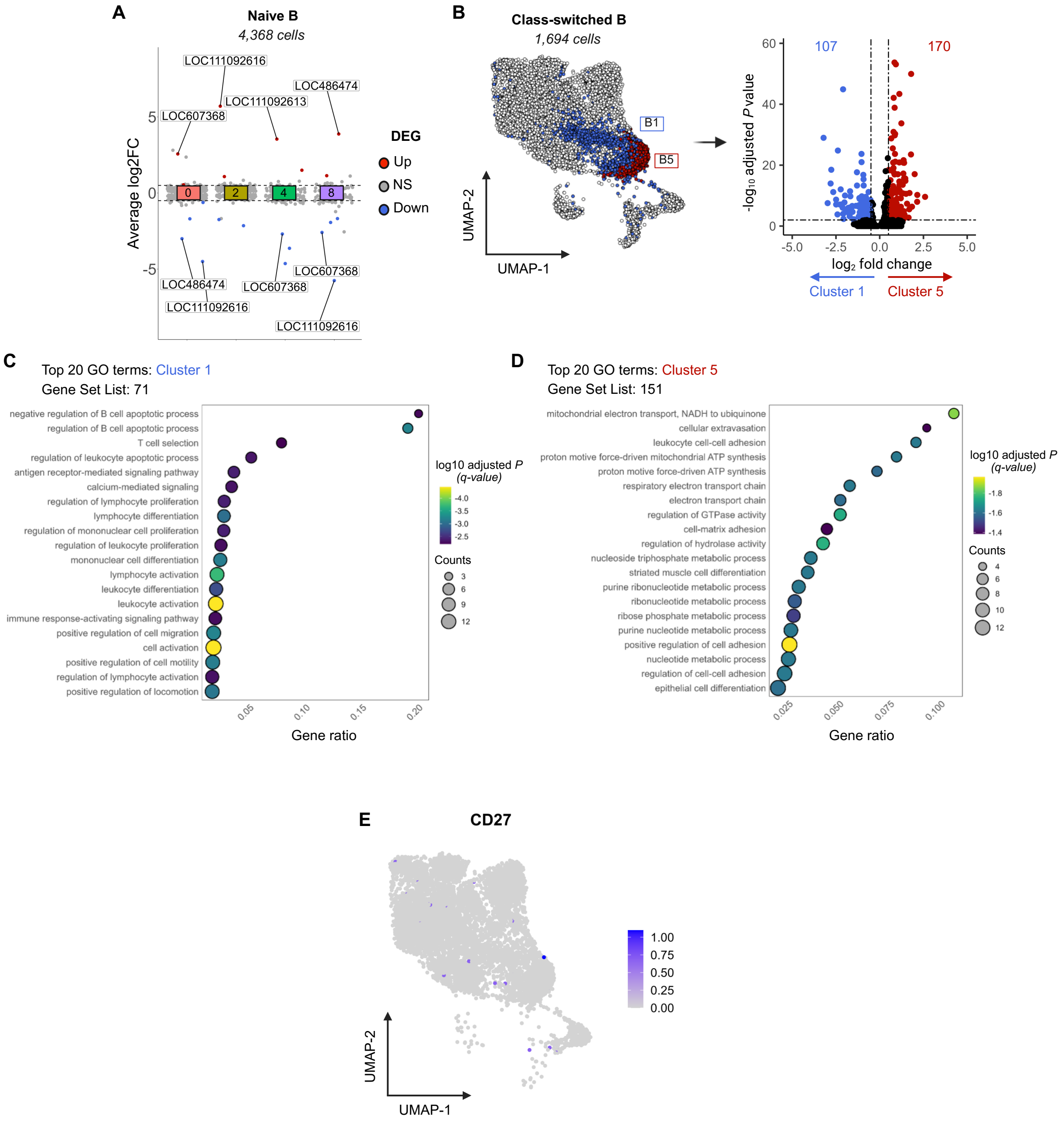

Figure S3: Transcriptional and functional divergence among similarly annotated B cell subsets. Related to Figure 2.
(A) Naive B cell subsets show minimal transcriptional differences and segregate according to the expression of specific immunoglobulin light chain genes. DEGs were defined by $|\log_2 FC| > 0.5$ and adjusted $P < 0.01$. DEG: differentially expressed genes; LOC486474: *IGLC7*-like; LOC607368: *IGLV3-1*; LOC111092616 and LOC111092613: *IGLL1*-like; NS: non-significant.
(B) UMAP and volcano plot highlighting transcriptional divergence between class-switched B cell subsets (1 (B_1) and 5 (B_2)). DEGs were defined by $|\log_2 FC| > 0.5$ and adjusted $P < 0.01$.
(C and D) Metascape[1] overrepresentation GO analysis of up- and downregulated genes between class-switched B cell subsets (5 vs 1). Downregulated genes (cluster 1, **(C)**) and upregulated genes (cluster 5 **(D)**) were analyzed for Biological Process enrichment ($|\log_2 FC| > 0.5$, adjusted $p < 0.01$), with the top 20 terms displayed by gene ratio and log10 adjusted *P*-value.
(E) Feature plot showing minimal *CD27* expression across major B cell subpopulations.

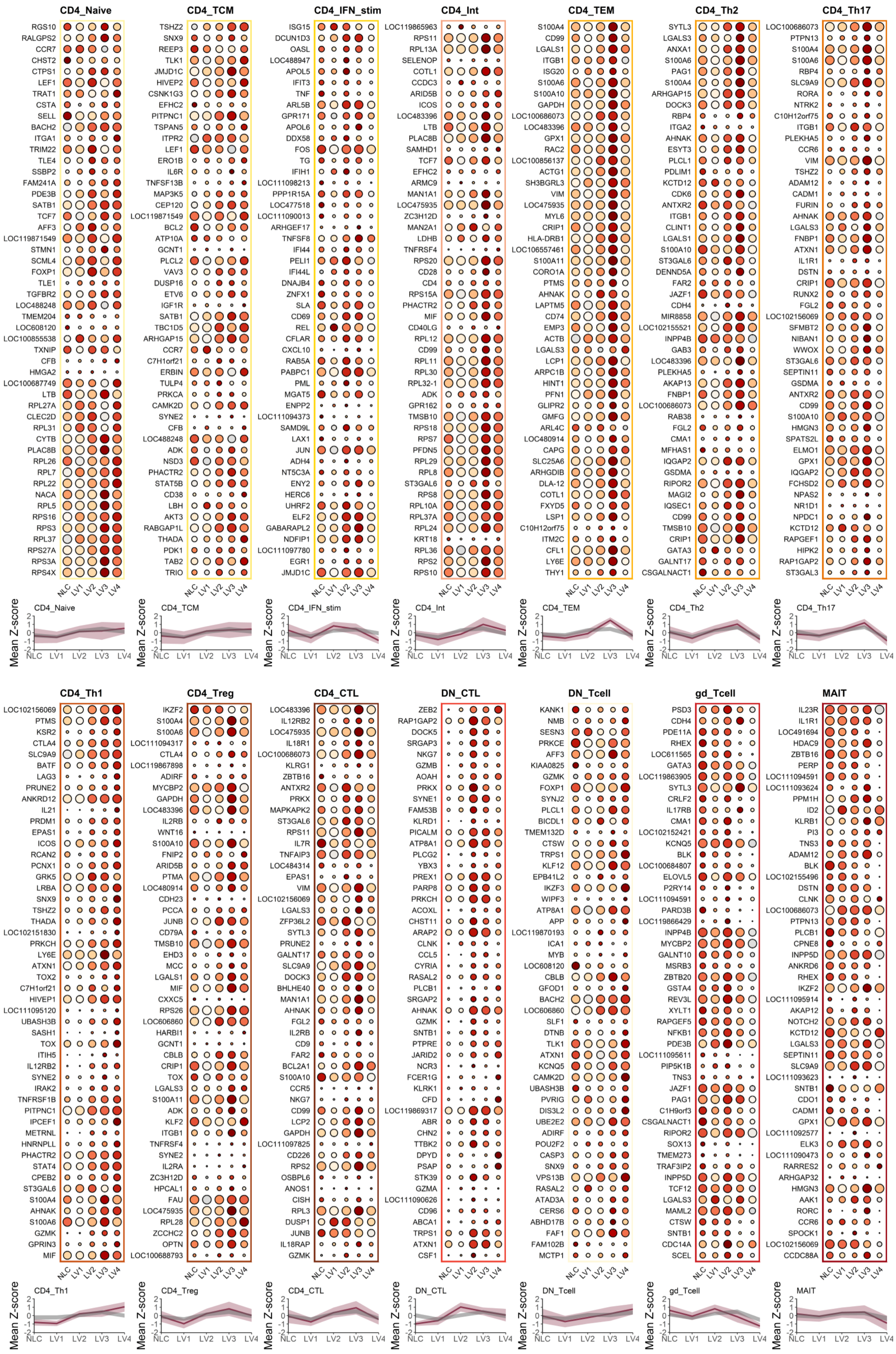

CD4_Naive
CD4_TCM
CD4_IFN_stim
CD4_Int
CD4_TEM
CD4_Th2
CD4_Th17
CD4_Th1
CD4_Treg
CD4_CTL
DN_CTL
DN_Tcell
gd_Tcell
MAIT
NLC LV1 LV2 LV3 LV4
Mean Z-score

Figure S4: Transcriptional signature dynamics of $CD4^{+}$ and DN T cells populations across CanL stages. Related to Figure 4.

Dot plots display gene expression and cell proportions across CanL clinical stages for each $CD4^{+}$/doublet-negative (DN) subset, indicated by their border colors. The dynamics of the top 50 DEGs defining the signature are summarized with line graphs showing the mean Z-score (dark wine-red line) and its standard deviation (light wine-red ribbon). These trajectories are visualized alongside a control distribution (grey ribbon) representing the upper and lower confidence intervals generated by bootstrapping (n = 100,000) non-signature genes.

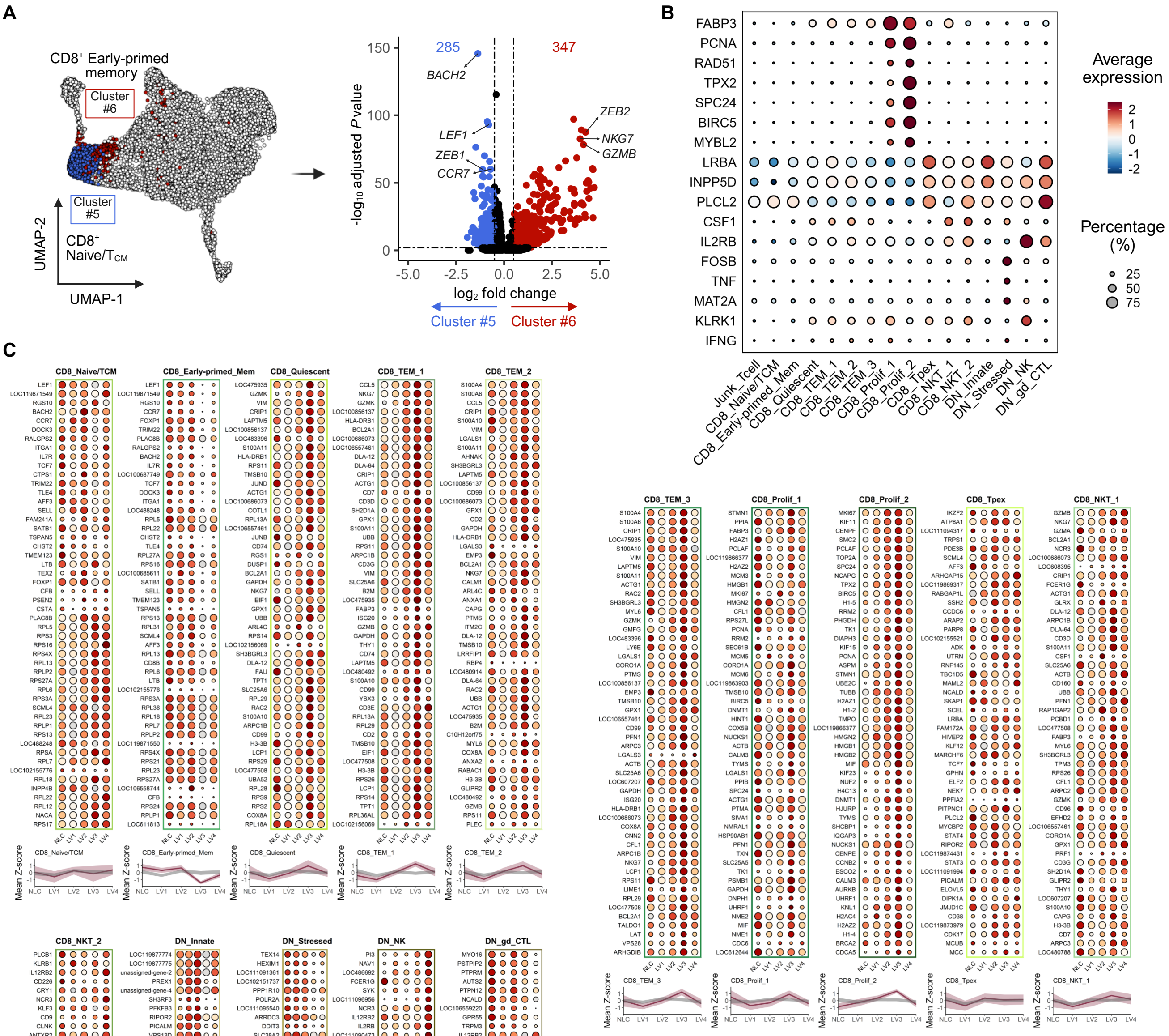

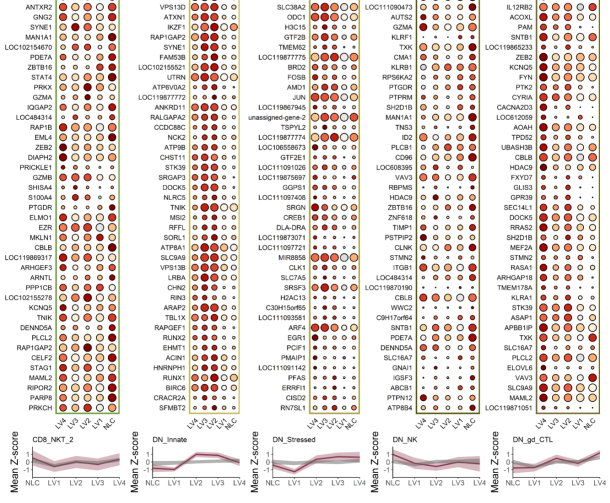

Figure S5: Differential expression, canonical marker profiles, and transcriptional signature dynamics of CD8+ and cytotoxic DN T/NK cell subsets across CanL stages. Related to Figure 5.

(A) Volcano plot showing differential expression between CD8+ Early-primed memory (cluster 6) and naïve/TCM (cluster 5) T cell subsets (|$\log_2$FC| > 0.5, adjusted P < 0.01).

(B) Dot plot showing additional canonical marker expression and proportions of annotated CD8+ and Cyto_DN subclusters.

(C) Dot plots display gene expression and cell proportions across CanL clinical stages for each CD8+/Cyto_DN subset, indicated by their border colors. The dynamics of the top 50 DEGs defining the signature are summarized with line graphs showing the mean Z-score (dark wine-red line) and its standard deviation (light wine-red ribbon). These trajectories are visualized alongside a control distribution (grey ribbon) representing the upper and lower confidence intervals generated by bootstrapping (n = 100,000) non-signature genes.

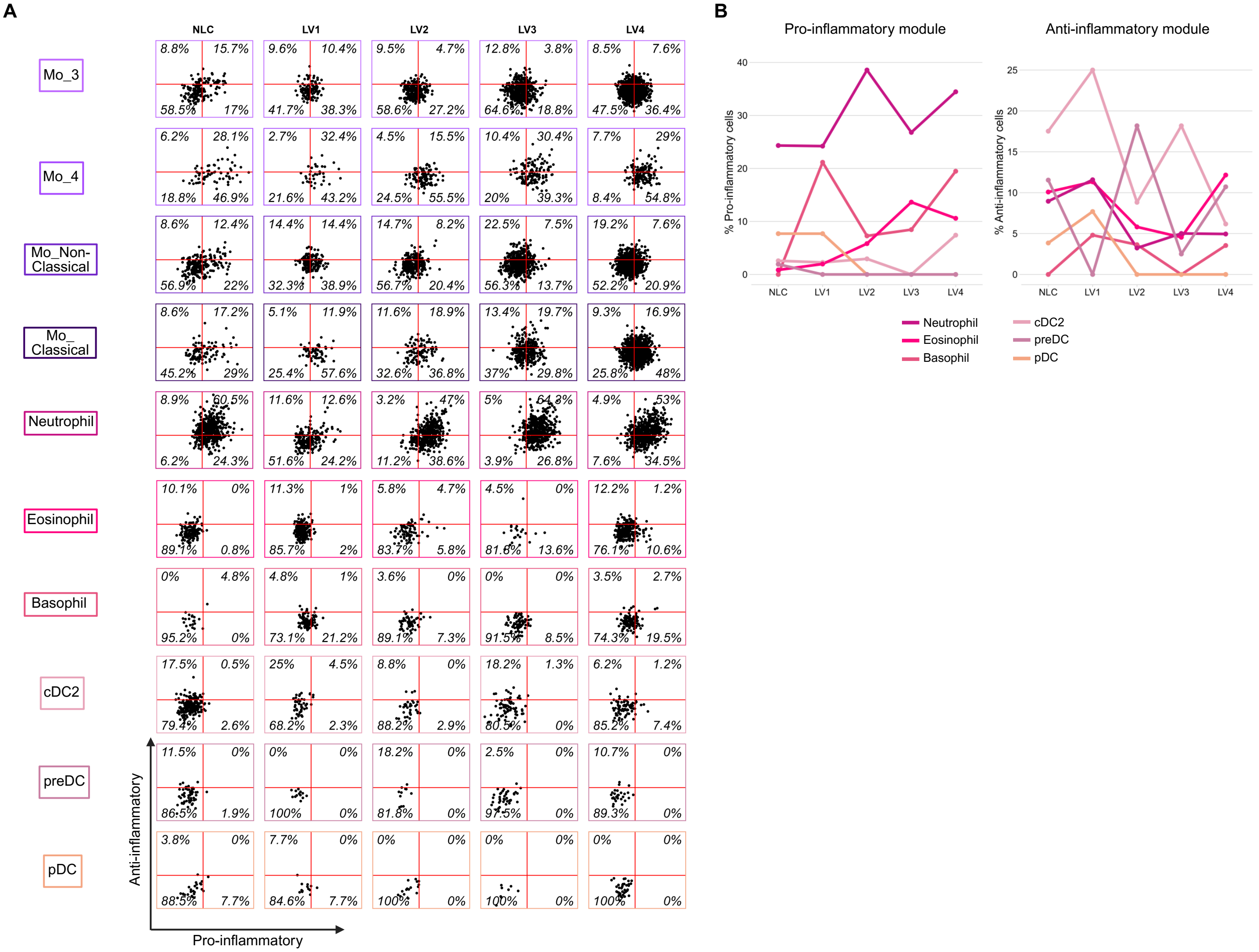

Figure S6: Inflammatory profiling of myeloid populations. Related to Figure 6.

(A) Scatter plots of inflammatory profiling that juxtapose pro- and anti- inflammatory module scores of Mo_3, Mo_4, Mo_Non-Classical, Mo_Classical, Neutrophil, Eosinophil, Basophil, cDC2, preDC and pDC cell populations at different clinical stages of CanL. Plots classify cells as pro-inflammatory (top left), anti-inflammatory (bottom right) non-inflammatory (bottom left) or mixed inflammatory (top right); and show percentage of cells in each quartile.

(B) Line graphs summarizing inflammatory profiling of Neutrophil, Eosinophil, Basophil, cDC2, preDC and pDC populations across CanL clinical stage. The graphs visualize percentages of cells classified as pro- or anti-inflammatory at different clinical stages of CanL.

Table S1. Cohort demographics and bloodwork. Overview of the age, sex, serum chemistry, and blood counts by LeishVet status.

	**Clinical stage**				
	**NLC**	**LV1**	**LV2**	**LV3**	**LV4**
	N = 3[a]	*N* = 2[b]	*N* = 6	*N* = 3	*N* = 2
Demographics					
Age (years)					
Range (%)					
Juvenile (0-2)	0	2 (100)	1 (16.67)	1 (33.33)	0
Adult (3-5)	2 (75)	0	4 (66.67)	1 (33.33)	2 (100)
Elderly (≥6)	0	0	1 (16.67)	1 (33.33)	0
Unknown	1 (25)	0	0	0	0
Mean (SD)	3.5 (0.707)	1 (0)	4.167 (1.329)	4.333 (2.082)	3.5 (0.707)
Sex (%)					
Male	1 (33.33)	1 (50)	5 (83.33)	2 (66.67)	2 (100)
Female	1 (33.33)	1 (50)	1 (16.67)	1 (33.33)	0
Unknown	1 (33.33)	0	0	0	0
Tick-serology exposure (%)					
No	0	0	3 (50)	1 (33.33)	0
Yes	0	0	3 (50)	2 (66.67)	2 (100)
Bloodwork					
Creatinine					
Mean (SD) (0.5-1.5 mg/dL)	N/A	1 (0)	0.633 (0.225)	1.467 (1.343)	**3.650 (1.061)**
BUN					
Mean (SD) (9-31 mg/dL)	N/A	20 (0)	13.0 (2.280)	**55.67 (69.06)**	**81.0 (26.87)**
Total protein					
Mean (SD) (5.5-7.5 g/dL)	N/A	5.9 (0)	**8.733 (1.209)**	**8.967 (0.451)**	**9.85 (2.192)**
Albumin					
Mean (SD) (2.7-3.9 g/dL)	N/A	3.2 (0)	**1.617 (0.688)**	**1.633 (0.305)**	**1.45 (0.212)**
Globulin					
Mean (SD) (2.5-4.0 g/dL)	N/A	2.7 (0)	**7.117 (1.663)**	**7.333 (0.473)**	**8.4 (2.404)**
ALB/GLOB ratio					
Mean (SD) (0.7-1.5)	N/A	1.2 (0)	**0.267 (0.225)**	**0.267 (0.0577)**	**0.15 (0.0707)**

ALT					
Mean (SD) (18-121 U/L)	N/A	22 (0)	46.17 (30.05)	**17.33 (1.155)**	24.50 (19.09)
ALP					
Mean (SD) (5-160 U/L)	N/A	55 (0)	56.17 (33.33)	34.33 (22.37)	28.50 (6.364)
RBCs					
Mean (SD) (5.39-8.70 M/µL)	N/A	6.46 (0)	6.014 (1.237)	**3.87 (1.023)**	**3.665 (0.46)**
Hematocrit					
Mean (SD) (38.3-56.5%)	N/A	45 (0)	**38.07 (10.60)**	**27.97 (7.184)**	**27.10 (1.131)**
Hemoglobin					
Mean (SD) (13.4-20.7 g/dL)	N/A	15.80 (0)	12.88 (3.640)	**8.733 (2.230)**	**8.6 (0.1414)**
WBCs					
Mean (SD) (4.9-17.6 K/µL)	N/A	10.7 (0)	**18.9 (3.552)**[c]	13.53 (6.332)	**18.65 (8.556)**
Retics					
Mean (SD) (10-110 K/µL)	N/A	45 (0)	53.80 (22.24)	50.67 (17.01)	24.50 (12.02)
Platelets					
Mean (SD) (143-448 K/µL)	N/A	229 (0)	233.5 (58.38)	194.3 (76.96)	**133 (0)**

Bolded values indicate the mean is outside of the normal reference range.
ALB: albumin; ALP: alkaline phosphatase; ALT: alanine transaminase; BUN: blood urea nitrogen; CREA: creatinine; GLOB: globulin; LV: LeishVet score; NLC: non-*Leishmania* control; RBC: red blood cells; Retics: reticulocytes; SD: standard deviation; WBC: white blood cells.
[a]Pooled-sample case considered as missing value (unknown), except for tick-borne exposure.
[b]Only one LV1 dog had a full blood chemistry panel performed.
[c]Complete WBC was available for five of the six LV2 dogs.

Table S2. Frequencies (%) of each PBMC, B, T/NK, $CD4^+$ & DN T, $CD8^+$ & cytotoxic DN T/NK, and myeloid population across clinical stages. Related to Figures 1-6.

**Population**	**Clinical stage**				
	**NLC**	**LV1**	**LV2**	**LV3**	**LV4**
PBMC (Level 1)					
T/NK	58.41	74.41	72.23	58.00	45.32
B	14.67	9.83	13.73	10.87	11.42
Myeloid	26.92	15.76	14.05	31.13	43.26
B cells (Level 2)					
Transitional	4.13	9.72	6.28	4.54	3.39
Naive	51.38	59.67	60.75	54.12	59.36
Unswitched	14.03	11.08	10.56	11.04	9.99
B_1	20.65	9.20	11.94	22.22	17.39
B_2	6.11	4.81	7.79	5.90	5.91
Plasma cells	3.70	5.54	2.68	2.19	3.96
T/NK cells (Level 2)					
$CD4^+$	64.82	56.91	34.20	34.50	44.75
$CD8^+$	20.50	26.38	50.54	49.33	38.87
DN	9.00	8.32	6.34	7.81	7.08
Cytotoxic DN	5.68	8.39	8.92	8.36	9.29
$CD4^+$ & DN T cells (Level 3)					
CD4_Naive	32.82	45.55	29.44	20.12	24.66
CD4_TCM	5.28	7.26	4.78	5.91	4.85
CD4_IFN_stim	1.83	2.86	2.68	3.43	2.21
CD4_Int	13.08	10.55	8.98	11.87	12.72
CD4_T_{EM}	14.08	8.01	11.67	12.58	10.51
CD4_T_H2	8.28	5.38	9.16	15.53	12.07
CD4_T_H17	3.83	3.03	3.33	6.64	8.03
CD4_T_H1	1.17	1.17	2.45	4.14	3.83
CD4_T_{REG}	3.39	1.80	2.14	2.79	2.43
CD4_CTL	5.11	3.62	3.87	4.49	3.85
DN_CTL	4.78	2.25	14.16	7.86	11.40
DN_Tcell	1.78	1.12	2.53	1.64	2.21
gd_Tcell	4.28	6.95	4.24	2.54	1.19
MAIT	0.31	0.45	0.58	0.45	0.05
$CD8^+$ & cytotoxic DN T/NK cells (Level 3)					
CD8_Naive/T_{CM}	10.58	10.78	5.00	4.17	7.20
CD8_Early-primed_Mem	13.58	9.84	5.21	1.60	3.54
CD8_Quiescent	18.75	15.44	15.01	12.76	15.21

CD8_TEM_1	9.42	13.83	19.82	22.14	19.68
CD8_TEM_2	2.42	4.11	5.99	5.65	4.91
CD8_TEM_3	6.33	4.94	3.68	4.70	3.75
CD8_Prolif_1	0.75	1.37	2.10	3.46	2.29
CD8_Prolif_2	2.42	1.25	1.27	2.29	2.14
CD8_T_{PEX}	9.42	7.76	11.00	10.64	8.43
CD8_NKT_1	6.67	7.64	9.12	8.87	9.47
CD8_NKT_2	5.25	5.49	8.73	10.33	6.01
DN_Innate	1.50	2.74	5.49	5.53	3.39
DN_Stressed	4.08	3.13	4.85	3.53	4.76
DN_NK	4.58	3.57	0.99	1.74	0.60
DN_gd_CTL	4.25	8.11	1.74	2.58	8.60
Myeloid cells (Level 2)					
Mo_1	13.55	13.48	18.18	17.39	21.55
Mo_2	12.27	15.73	19.96	21.26	18.49
Mo_3	8.13	8.61	11.16	13.27	14.00
Mo_4	3.27	2.77	5.29	3.96	2.33
Mo_Non-Classical	10.69	12.51	11.78	15.27	12.56
Mo_Classical	4.75	4.42	4.57	8.96	14.89
Neutrophil	26.28	14.23	19.34	13.48	8.49
Eosinophil	6.08	15.21	4.14	0.65	3.83
Basophil	1.07	7.79	2.65	2.09	1.70
cDC2	9.92	3.30	1.64	2.26	1.22
preDC	2.66	0.97	0.53	1.17	0.42
pDC	1.33	0.97	0.77	0.23	0.53

cDC: conventional dendritic cell; CM: central memory; CTL: cytotoxic T lymphocyte; DN: double-negative; EM: effector memory; gd: gamma-delta; pDC: plasmacytoid dendritic cell; preDC: precursor dendritic cell; NLC: non-*Leishmania* control; MAIT: mucosal associated-invariant T; Mo: monocyte; NK: Natural Killer; PBMC: peripheral blood mononuclear cells; PEX: progenitor exhaustion.

Table S3. Fold-changes in log odds ratios comparing abundances of peripheral immune cell types and major T/NK cell subsets across CanL stages versus NLC. Related to Figures 1 and 3.

Contrast	LV score	*Z* ratio	FC OR	CI	Adjusted *P*
PBMC (Level 1)					
T/NK : Myeloid	LV1	29.69	4.0760844	[3.72, 4.47]	1.26e-193
T/NK : Myeloid	LV2	34.00	4.1735447	[3.84, 4.53]	9.54e-253
T/NK : Myeloid	LV3	-5.33	0.801078	[0.74, 0.87]	9.98e-08
T/NK : Myeloid	LV4	-31.90	0.2850927	[0.26, 0.31]	6.20e-223
T/NK : B	LV1	21.90	3.2650699	[2.94, 3.63]	1.07e-105
T/NK : B	LV2	15.11	2.0019679	[1.83, 2.19]	2.84e-51
T/NK : B	LV3	6.54	1.3867489	[1.26, 1.53]	8.32e-11
T/NK : B	LV4	-5.07	0.7871008	[0.72, 0.86]	4.00e-07
Myeloid : B	LV1	-3.90	0.801031	[0.72, 0.90]	9.74e-05
Myeloid : B	LV2	-15.01	0.479681	[0.44, 0.53]	1.36e-50
Myeloid : B	LV3	10.65	1.7311035	[1.56, 1.92]	2.36e-26
Myeloid : B	LV4	20.97	2.7608586	[2.51, 3.04]	4.63e-97
T/NK cells (Level 2)					
$CD4^+$: $CD8^+$	LV1	-11.46	0.51577560	[0.46, 0.58]	1.98e-30
$CD4^+$: $CD8^+$	LV2	-49.99	0.07121129	[0.06, 0.08]	0.00e+00
$CD4^+$: $CD8^+$	LV3	-44.92	0.07572185	[0.07, 0.08]	0.00e+00
$CD4^+$: $CD8^+$	LV4	-29.98	0.17826158	[0.16, 0.20]	2.70e-197
$CD4^+$: DN	LV1	-3.31	0.7805136	[0.67, 0.90]	0.0009
$CD4^+$: DN	LV2	-12.52	0.4123549	[0.36, 0.47]	1.16e-35
$CD4^+$: DN	LV3	-14.30	0.3338367	[0.29, 0.39]	9.29e-46
$CD4^+$: DN	LV4	-7.19	0.5704599	[0.49, 0.66]	8.46e-13
$CD4^+$: Cytotoxic DN	LV1	-9.05	0.4712033	[0.40, 0.55]	1.39e-19
$CD4^+$: Cytotoxic DN	LV2	-22.64	0.1733844	[0.15, 0.20]	6.57e-113
$CD4^+$: Cytotoxic DN	LV3	-19.80	0.1885554	[0.16, 0.22]	5.79e-87
$CD4^+$: Cytotoxic DN	LV4	-16.23	0.2584756	[0.22, 0.30]	4.13e-59
$CD8^+$: DN	LV1	5.31	1.513281	[1.30, 1.76]	1.11e-07
$CD8^+$: DN	LV2	24.05	5.790584	[5.02, 6.68]	3.25e-127
$CD8^+$: DN	LV3	18.86	4.408724	[3.78, 5.14]	5.27e-79
$CD8^+$: DN	LV4	14.46	3.200128	[2.73, 3.75]	2.92e-47

CD8$^+$: Cytotoxic DN	LV1	-1.05	0.913582	[0.77, 1.08]	0.2935
CD8$^+$: Cytotoxic DN	LV2	11.20	2.434788	[2.08, 2.85]	1.66e-28
CD8$^+$: Cytotoxic DN	LV3	10.61	2.490106	[2.10, 2.95]	5.62e-26
CD8$^+$: Cytotoxic DN	LV4	4.34	1.449980	[1.23, 1.71]	1.90e-05
DN : Cytotoxic DN	LV1	-5.13	0.6037093	[0.50, 0.73]	2.85e-07
DN : Cytotoxic DN	LV2	-9.38	0.4204737	[0.35, 0.50]	2.59e-20
DN : Cytotoxic DN	LV3	-5.72	0.5648133	[0.46, 0.69]	1.46e-08
DN : Cytotoxic DN	LV4	-7.87	0.4531005	[0.37, 0.55]	6.94e-15

DN: double-negative; LV: LeishVet; NLC: non-*Leishmania* control; PBMC: peripheral blood mononuclear cells.

SUPPLEMENTAL REFERENCE

1. Zhou, Y., Zhou, B., Pache, L., Chang, M., Khodabakhshi, A.H., Tanaseichuk, O., Benner, C., and Chanda, S.K. (2019). Metascape provides a biologist-oriented resource for the analysis of systems-level datasets. Nat Commun *10*, 1523. 10.1038/s41467-019-09234-6
